# Human Case of *Streptococcus suis* Serotype 16 Infection

**DOI:** 10.3201/eid1401.070534

**Published:** 2008-01

**Authors:** Ho Dang Trung Nghia, Ngo Thi Hoa, Le Dieu Linh, James Campbell, To Song Diep, Nguyen Van Vinh Chau, Nguyen Thi Hoang Mai, Tran Tinh Hien, Brian Spratt, Jeremy Farrar, Constance Schultsz

**Affiliations:** * Oxford University Clinical Research Unit, Ho Chi Minh City, Vietnam; † Hospital for Tropical Diseases, Ho Chi Minh City, Vietnam; ‡ Imperial College London, London, United Kingdom

**Keywords:** Streptococcus suis, serotype 16, typing, dispatch

## Abstract

*Streptococcus suis* infection is an emerging zoonosis in Southeast Asia. We report a fatal case of *S. suis* serotype 16 infection in a Vietnamese man in 2001.

*Streptococcus suis* is a gram-positive, facultatively anaerobic coccus that may cause pneumonia, meningitis, septicemia, and arthritis in pigs. The pig can also be a healthy carrier of *S. suis* in the upper respiratory tract (particularly the tonsils and nasal cavities), the genital tract, and the alimentary tract (*1*,*2*). The first case of *S. suis* infection in humans was reported in Denmark in 1968. Since then, increasing numbers of cases have been reported in many countries, including the Netherlands; the United Kingdom; France; Hong Kong Special Administrative Region, People’s Republic of China; Thailand; Taiwan; and the United States (*3*–*7*). A recent outbreak of *S. suis* infections in Sichuan Province, People’s Republic of China, emphasized the importance of *S. suis* as an emerging zoonosis (*8*). *S*. *suis* is also the most important cause of bacterial meningitis in adults at the Hospital for Tropical Diseases (HTD) in Ho Chi Minh City, Vietnam (N.T.H Mai et al., unpub. data). The number of cases is likely underreported and will likely increase further with increased awareness and enhanced capacity to culture and identify *S. suis*.

## The Patient

A 57-year-old unemployed man from Long An Province, southern Vietnam, who had a history of alcohol abuse, had a 10-day history of abdominal pain, jaundice, anorexia, and weight loss. At the time of admission to HTD in 2001, the patient was lethargic, his vital signs were stable, and his neck was not stiff. Physical examination showed cutaneous spider naevi, jaundice, hepatosplenomegaly, and ascites. Leukocyte count was 15.3 ×10^3^ cells/μL (70% neutrophils), blood urea nitrogen 12.1 mmol/L (reference range 3.57–7.14 mmol/L), creatinine 150 μmol/L (<115 mmol/L), sodium 127 mmol/L (135–145 mmol/L), potassium 6.67 mmol/L (3.5–5.1 mmol/L), serum aspartate aminotransferase 87 IU/L (12–30 IU/L), serum alamine aminotransferase 41 IU/L (13–40 IU/L), and albumin 20 g/L (35–52 g/L). An abdominal ultrasound examination showed hepatosplenomegaly and ascites. Ascitic fluid was cloudy and contained 5 g/L protein. Results of Gram stain and culture of ascitic fluid were negative. A diagnosis of spontaneous bacterial peritonitis associated with alcoholic liver cirrhosis was made, and the patient was treated with 2 g/day of ceftriaxone.

Twenty-four hours after admission, acute respiratory distress developed. The patient’s family decided to take him home because they were unable to pay for further treatment; the patient died on the same day. The blood culture (BACTEC 9050 system; Becton Dickinson Microbiology Systems, Sparks, MD, USA), which was taken at the time of admission, grew *S. suis* 24 hours after collection. Further inquiries into potential pig exposure, after the blood culture results were reported, indicated that the patient kept pigs near his house and was known to regularly consume portions of the pig that had a high risk of being contaminated, such as the intestine.

*S. suis* was identified on the basis of colony morphology, negative katalase reaction, optochin resistance, and APIStrep (bioMérieux, Marcy l’Etoile, France). Serotyping was performed by slide agglutination using specific antisera (Statens Serum Institute, Copenhagen, Denmark) and was positive for serotype 16. Confirmation of the serotype was performed at the International Reference Laboratory for *S. suis* Serotyping, Quebec City, Quebec, Canada (M. Gottschalk). This strain was susceptible to penicillin (MIC 0.032 mg/L), ceftriaxone (0.064 mg/L), rifampin (0.032 mg/L), chloramphenicol (2 mg/L), erythromycin (0.064 mg/L), levofloxacin (0.38 mg/L), and vancomycin (0.5 mg/L) but resistant to tetracycline (64 mg/L) by Etest (AB-Biodisk, Solna, Sweden) when Clinical Laboratory Standard Institute breakpoints were used. On pulsed-field gel electrophoresis (PFGE) that used restriction enzyme *Sma*I, this strain showed little similarity with a representative set of serotype 2 isolates from Vietnam (Figure). Multilocus sequence typing (MLST) (www.mlst.net) showed that the sequence of 5 of 7 alleles of the included housekeeping genes had not been previously described. Thus, this strain was assigned the new sequence type 106. On eBURST analysis (www.mlst.net), this sequence type does not belong to any of the clonal complexes but is a singleton. PCR for detection of the genes encoding the putative virulence factors extracellular protein factor (EF) and suilysin were negative. Results of Western blot for detection of muramidase-released protein (MRP) and EF, using rabbit polyclonal antibody against MRP and EF (provided by H. Smith, the Netherlands), were also negative. S. suis serotype 2 strains 31533 and 89-1591 (provided by M. Gottschalk, Canada) were used as positive and negative controls, respectively.

**Figure:**
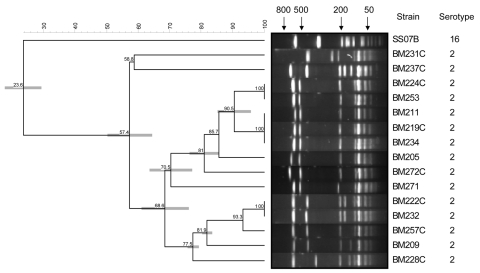
Pulsed-field gel electrophoresis after *Sma*I digestion of *Streptococcus suis* serotype 16 strain SS07 and a representative set of *S. suis* serotype 2 strains isolated from patients with meningitis in southern Vietnam. A dendrogram was generated by Dice analysis (optimization 0.5%, band tolerance 1%) and cluster analysis with unweighted pair group method with arithmetic average, using Bionumerics software (Applied Maths, Sint-Martens-Latem, Belgium). Numbers in dendrogram indicate percentage of similarity. Arrow numbers indicate molecular size (kb).

The total number of human *S. suis* infections reported until August 2006 was ≈400, and nearly 90% of these cases occurred in China, Thailand, Hong Kong, Taiwan, and the Netherlands (*4*). These data did not include at least 200 cases of *S. suis* infection in Vietnam (C. Schultsz, unpub. data). At present, 33 capsular serotypes of *S. suis* have been recognized (*1*,*4*). *S. suis* serotype 2 is considered to be the most pathogenic to pigs and humans. All human cases of *S. suis* infection for which serotyping was available were caused by *S. suis* serotype 2, except for 1 case of serotype 1 (*9*), 1 case of serotype 4 (*3*), and 1 case of serotype 14 (*6*). Among 116 cases of *S. suis* meningitis seen at HTD from 1997 through 2005, 115 were caused by serotype 2 and 1 by serotype 14.

*S. suis* serotype 2 can cause meningitis, septicemia and septic shock, arthritis, endocarditis, pneumonia, endophthalmitis, and cellulitis in humans (*3*,*5*,*8*). The mortality rate for *S. suis* serotype 2 meningitis is <10% (*10*), but it can reach 70% among patients with septicemia and septic shock (*11*). The exact route of infection for humans is not known. Cases have been linked to accidental inoculation through skin injuries, for example, during occupational exposure to pigs and pork, but inhalation of aerosols and ingestion of contaminated food have also been suggested (*3*,^9^,*11**,**12*). Preexisting medical conditions, such as alcoholism and liver cirrhosis, as was present in our patient, or a prior splenectomy may predispose to severe infection.

Serotype 16 has never been isolated from humans and, to our knowledge, has also rarely been reported as a cause of invasive disease in pigs. One isolate of *S. suis* serotype 16 was reported from a diseased pig in Germany and 4 isolates from slaughter pigs in South Korea (*13**,**14*). The *S. suis* serotype 16 strain was sensitive to all antimicrobial agents tested except tetracycline, as has been reported for serotype 2 isolates (*5*). PFGE results showed that this human serotype 16 isolate was unrelated to human serotype 2 isolates. On MLST, this isolate had a new sequence type that did not belong to any of the clonal complexes. In contrast, most serotype 2 isolates reported so far belong to clonal complex 1 (www.mlst.net). Taken together, these results suggest that capsule switch, such as has been observed for *S. pneumoniae,* does not explain the emergence of invasive isolates of a different serotype. In addition, the PCR and Western blot analyses indicate that the serotype 2 capsule, EF, MRP, or suilysin is not required for virulence of *S. suis* in humans, as has also been shown in pigs.

## Conclusions

*S. suis* infection is an emerging zoonosis in Asia. Strains with serotype 16 are among those capable of infecting humans.
